# Effect of influenza virus infection on key metabolic enzyme activities in MDCK cells

**DOI:** 10.1186/1753-6561-5-S8-P129

**Published:** 2011-11-22

**Authors:** Robert Janke, Yvonne Genzel, Maria Wetzel, Udo Reichl

**Affiliations:** 1 Max Planck Institute for Dynamics of Complex Technical Systems, Bioprocess Engineering group, Sandtorstraße 1, 39106 Magdeburg, Germany; 2 Chair for Bioprocess Engineering, Otto von Guericke University of Magdeburg, Universitätsplatz 2, 39106 Magdeburg, Germany

## Background

Influenza, or “flu”, is an upper respiratory tract infection caused by a virus belonging to the family of *Orthomyxoviridae*. Influenza can pose a serious risk to the health of mainly the elderly, the very young, and to people suffering from medical conditions (e.g. weak immune system). For example, seasonal influenza strains are fatal to more than 50,000 people annually in the United States alone [1]. The most effective measure for preventing influenza-related morbidity and mortality is annual vaccination. Seasonal influenza vaccines are almost exclusively produced using the traditional egg-based manufacturing process. However, the main limitation of egg-based technology (especially in the case of a pandemic) is the time-consuming production process (~6 months). Furthermore, people with serious egg allergy cannot be vaccinated when trace amounts of egg protein remain in the final formulation. Therefore, new production processes using continuous cell lines for influenza vaccine manufacturing are currently being established [2].

Influenza viruses take advantage of the host cell metabolism to replicate their genetic material and to synthesize viral proteins. The influenza virus particle consists of three major parts: the ribonucleocapsid, the matrix protein M1, and the envelope, which is derived from the plasma membrane of the host cell. The lipid bilayer contains the ion channel protein M2 and the immunogenic glycoproteins hemagglutinin and neuraminidase [3]. The replication cycle of influenza viruses including entry, uncoating, genome transcription and replication, assembly and release has been studied extensively with type A strains [4]. So far, only few studies have characterized the influence of influenza infection on the central carbon metabolism of host cells [5]. Madin-Darby canine kidney (MDCK) cells are considered a suitable substrate for cell culture-based influenza vaccine manufacturing [2,6]. In this study, key metabolic enzyme activities were analyzed in MDCK cells infected with an influenza A virus strain to improve our understanding of virus-host cell interaction and cell response.

## Materials and methods

All chemicals and enzymes were purchased from Sigma (Taufkirchen, Germany) or Roche (Mannheim, Germany). Adherent MDCK cells obtained from the ECACC (No. 84121903) were routinely cultured in 6-well plates containing 4 mL of GMEM-based medium (2 mM glutamine, 30 mM glucose, 10 % (v/v) fetal calf serum, 2 g/L peptone, 48 mM NaHCO_3_) in a CO_2_ incubator at 37 °C and 5 % CO_2_ to the stationary phase (~5 days of growth, 4.0-4.2 x 10^6^ cells) [7]. Cell concentration and viability was determined for samples from 6-well plates as described previously [8]. MDCK cells were either mock-infected or infected with MDCK cell-adapted human influenza virus A/Puerto Rico/8/34 (H1N1) from the Robert Koch Institute (Berlin, Germany) at a multiplicity of infection of 20 as described previously [5,9]. Cells were washed twice with ice-cold phosphate-buffered saline 9 hours post infection (hpi), and the complete plate was then snap-frozen in liquid nitrogen and stored at -80 °C until further use. After thawing, samples were extracted by sonification on maximum power for 30 s with 1 mL extraction buffer [7] and kept at 0–4 °C. To remove cell debris, samples were centrifuged at 16,000 x g for 5 min. The supernatant was used to measure the respective enzyme activities. The procedure for the enzyme activity analysis and the details for the specific assay mixes were as described previously [7].

## Results

**Figure 1:**
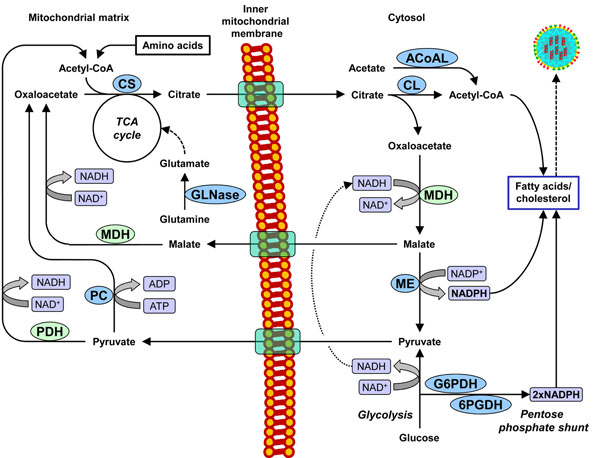
Reaction network scheme of the central carbon metabolism of adherent MDCK cells. Enzymes up-regulated in influenza A (H1N1) infected cells are highlighted in blue. Abbreviations: 6PGDH, 6-phosphogluconate dehydrogenase; ACoAL, acetate-CoA ligase; CL, citrate lyase; CS, citrate synthase; G6PDH, glucose-6-phosphate dehydrogenase; GLNase, glutaminase; MDH, malate dehydrogenase; ME, malic enzyme; PDH, pyruvate dehydrogenase; PC, pyruvate carboxylase.

The maximum catalytic activities of 28 enzymes from central carbon metabolism were measured under substrate saturation using a recently developed assay platform for mammalian cells [7]. Table 1 shows the maximum metabolic enzyme activities in mock-infected and influenza A (H1N1) infected MDCK cells. The activities of different enzymes comprise several orders of magnitude. The overall range covers values from 0.24±0.10 nmol/min/10^6^ cells (pyruvate dehydrogenase, PDH) to 10348.06±1663.65 nmol/min/10^6^ cells (triose-phosphate isomerase, TPI) in H1N1 and mock-infected cells, respectively. Highest activities (>1000 nmol/min/10^6^ cells) were found for TPI, pyruvate kinase, lactate dehydrogenase, and malate dehydrogenase, while the other activities were in the range of 1 to 500 nmol/min/10^6^ cells. Very low enzyme activities, which indicate possible rate-limiting steps in the respective metabolic pathway, were found for PDH, pyruvate carboxylase (PC), NAD^+^-dependent isocitrate dehydrogenase (NAD-ICDH), and glutamine synthetase (<1 nmol/min/10^6^ cells). Significant differences in catalytic activity between infected cells and mock-infected MDCK cells were found for 9 enzymes: glucose 6-phosphate dehydrogenase and 6-phosphogluconate dehydrogenase from the pentose phosphate pathway, glutaminase and malic enzyme from glutaminolysis, PC, citrate synthase, citrate lyase and NAD-ICDH from the citric acid cycle, and acetate-CoA-ligase (Figure 1). The fact that maximum enzyme activities in H1N1 infected cells were always higher than in mock-infected cells suggests an up-regulation of metabolic activities during early virus replication.

**Table 1:** Maximum enzyme activities of glycolysis, pentose phosphate pathway, TCA cycle, and glutaminolysis in MDCK cells infected with influenza A (H1N1) compared to mock-infected cells.

			Enzyme activities in adherent MDCK cells^a^ (nmol/min/10^6^ cells)							
	Enzyme	EC number	Mock-infected				H1N1 infected			

*Glycolysis*										
	Hexokinase	2.7.1.1	21.80	±	3.95		21.89	±	1.48	
	Phosphoglucose isomerase	5.3.1.9	465.12	±	73.94		456.93	±	111.90	
	Phosphofructokinase	2.7.1.11	29.28	±	6.14		30.56	±	2.08	
	Fructose-1,6-bisphosphate aldolase	4.1.2.13	23.62	±	3.32		20.74	±	8.41	
	Triose-phosphate isomerase	5.3.1.1	10348.06	±	1663.65		10148.62	±	698.01	
	Glyceraldehyde-3-phosphate dehydrogenase	1.2.1.12	412.90	±	73.27		413.61	±	31.61	
	Pyruvate kinase	2.7.1.40	1004.26	±	43.53		1001.92	±	112.95	
	Lactate dehydrogenase	1.1.1.27	1266.97	±	134.69		1302.10	±	107.37	

*Pentose phosphate pathway*										
	Glucose-6-phosphate dehydrogenase	1.1.1.49	51.91	±	1.10		62.71	±	4.59	^b^
	6-phosphogluconate dehydrogenase	1.1.1.44	30.61	±	5.03		38.13	±	1.05	^b^
	Transketolase	2.2.1.1	18.63	±	6.65		19.19	±	2.52	
	Transaldolase	2.2.1.2	23.11	±	5.84		23.23	±	6.30	

*Tricarboxylic acid cycle*										
	Pyruvate dehydrogenase	1.2.4.1	0.30	±	0.07		0.24	±	0.10	
	Pyruvate carboxylase	6.4.1.1	0.48	±	0.11		0.82	±	0.23	^b^
	Citrate synthase	2.3.3.1	21.55	±	1.70		25.40	±	2.84	^b^
	Citrate lyase	2.3.3.8	4.48	±	0.55		6.00	±	0.98	^b^
	NAD^+^-linked isocitrate dehydrogenase	1.1.1.41	0.27	±	0.02		0.34	±	0.05	^b^
	NADP^+^-linked isocitrate dehydrogenase	1.1.1.42	44.27	±	2.73		44.57	±	5.37	
	Fumarase	4.2.1.2	74.62	±	6.56		77.85	±	11.75	
	Malate dehydrogenase	1.1.1.37	1115.42	±	90.90		1158.66	±	145.10	

*Glutaminolysis*										
	Glutaminase	3.5.1.2	3.29	±	0.29		3.90	±	0.34	^b^
	Glutamine synthetase	6.3.1.2	0.68	±	0.25		0.80	±	0.25	
	Glutamate dehydrogenase	1.4.1.2	2.54	±	0.51		2.20	±	0.31	
	Alanine transaminase	2.6.1.2	3.21	±	0.50		3.31	±	0.03	
	Aspartate transaminase	2.6.1.1	78.67	±	5.36		79.12	±	13.17	
	Malic enzyme	1.1.1.40	7.61	±	0.61		9.87	±	0.62	^b^
	Phosphoenolpyruvate carboxykinase	4.1.1.32	138.64	±	15.33		131.48	±	11.42	

*Miscellaneous*										
	Acetate-CoA ligase	6.2.1.1	2.01	±	0.13		2.69	±	0.34	^b^

^a^ Mean values and 95 % confidence intervals for three biological replicates.

^b^ Significantly different from mock-infected cells as determined by Student's *t*-test (p ≤ 0.05).

## Conclusions

In the present work, the effect of an influenza virus infection on host cell metabolism was investigated. Experimental data clearly suggests a change in metabolism of cells infected with human influenza A (H1N1), i.e. an up-regulation of key metabolic enzyme activities in MDCK cells. This shift in metabolism is most likely required to compensate for the metabolic imbalance caused by viral replication. Interestingly, activity of some key enzymes producing NADPH and acetyl-CoA, a precursor needed for lipid and cholesterol biosynthesis, were increased by at least 20 % in infected MDCK cells. Hence, fatty acid synthesis might play a crucial role for the replication of influenza viruses as they acquire lipid envelopes from their host cells.
